# A multi-provincial *Salmonella* Typhimurium outbreak in Canada associated with exposure to pet hedgehogs, 2017–2020

**DOI:** 10.14745/ccdr.v48i06a06

**Published:** 2022-06-09

**Authors:** Katharine Fagan-Garcia, Leann Denich, Joanne Tataryn, Rachelle Janicki, Olivia Van Osch, Ashley Kearney, Cynthia Misfeldt, Celine Nadon, Colette Gaulin, Victor Mah, Raminderjeet Sandhu, Michelle Waltenburg, Bijay Adhikari, Hanan Smadi, Anne-Marie Lowe

**Affiliations:** 1 Canadian Field Epidemiology Program, Public Health Agency of Canada, Toronto, ON; 2 Centre for Food-borne, Environmental and Zoonotic Infectious Diseases, Public Health Agency of Canada, Guelph, ON; 3 Centre for Food-borne, Environmental and Zoonotic Infectious Diseases, Public Health Agency of Canada, Saskatoon, SK; 4 National Microbiology Laboratory, Public Health Agency of Canada, Winnipeg, MB; 5 Direction de la vigie sanitaire, Ministère de la Santé et des Services sociaux, Québec City, QC; 6 Alberta Health, Edmonton, AB; 7 Alberta Health Services, Calgary, AB; 8 Division of Foodborne, Waterborne, and Environmental Diseases, Centers for Disease Control and Prevention, Atlanta, GA; 9 Government of Saskatchewan, Regina, SK; 10 Public Health New Brunswick, Fredericton, NB

**Keywords:** salmonella, *S.* Typhimurium, hedgehog, zoonotic, enteric, outbreak

## Abstract

**Background:**

In October 2020, an investigation began in Canada on an outbreak of *Salmonella* Typhimurium infections of the same strain as a concomitant outbreak in the United States (US) that was linked to pet hedgehogs. The objective of this article is to identify the source of the outbreak, determine if there was a link between the Canadian and US outbreaks and identify risk factors for infection to inform public health interventions.

**Methods:**

Cases were identified through whole genome sequencing of *S.* Typhimurium isolates. Information was collected on case exposures, including animal contact. Hedgehog and environmental specimens were tested for *S.* Typhimurium and a trace back investigation was conducted.

**Results:**

There were 31 cases in six provinces, with illness onset dates from June 1, 2017, to October 15, 2020. Median case age was 20 years and 52% were female. Isolates grouped together between 0–46 whole genome multi locus sequence typing allele differences. Of 23 cases with available exposure information, 19 (83%) reported contact with hedgehogs in the seven days prior to symptoms; 15/18 (83%) reported direct contact and 3/18 (17%) reported indirect contact. Trace back investigation did not identify a common source of hedgehogs but uncovered an industry with a complex distribution network. The outbreak strain was detected in samples collected from a hedgehog in one case’s home and from a hedgehog in a Québec zoo.

**Conclusion:**

Direct and indirect contact with hedgehogs was identified as the source of this *S.* Typhimurium outbreak. Public health communications aimed to increase awareness about the risks of zoonoses from hedgehogs and shared key hygienic practices to reduce disease transmission.

## Introduction

*Salmonella* remains a leading cause of human enteric illness in Canada. Symptoms of salmonellosis typically begin 6 to 72 hours after exposure, and can include fever, chills, diarrhea, abdominal cramps, headache, nausea and vomiting that usually end within 4–7 days ((1)). Although many infections are linked to consumption of contaminated foods, an estimated 13%–19% are associated with animal contact ((2–4)). *Salmonella* bacteria colonize the gastrointestinal tract of a wide range of host species; animals can experience clinical disease following infection, but most often no clinical signs are observed, with intermittent fecal shedding and carriage ((5)). Many *Salmonella* Typhimurium outbreaks in the United States (US) and Canada have been linked to direct or indirect contact with a variety of pets and their foods, including rodents and other small mammals (mice, rats, guinea pigs, hedgehogs), reptiles and amphibians (frogs, turtles, snakes) and dogs and cats ((6–8)).

Hedgehogs have gained popularity as pets in recent decades, with the African pygmy hedgehog (*Atelerix albiventris*) the species most often sold in the North American pet trade ((9–11)). Captive breeding is in place in Canada and the US, as importation directly from Africa is prohibited due to their potential to carry serious diseases including foot-and-mouth disease ((10–12)). Hedgehogs can be a source of several zoonotic diseases, including salmonellosis ((11,13,14)). *Salmonella* infections in hedgehogs can result in clinical illness; however, many remain asymptomatic carriers with prevalence of *Salmonella* carriage in wild hedgehog populations ranging from 0% to 96% ((10,11,14–16)).

A number of *Salmonella* outbreaks and individual cases linked to pet or wild hedgehogs have been reported since the 1990s ((11)), involving different serotypes including *S.* Tilene ((17–19)), *S.* Typhimurium ((10,16,19,20)), *S.* Enteritidis ((21,22)) and *S.* Stanley ((23)). In Canada in 1995–1997, there was a multi-provincial outbreak of 10 cases of *S.* Tilene associated with pet hedgehogs and sugar gliders ((18)). The US Centers for Disease Control and Prevention (CDC) investigated three multistate outbreaks of *S.* Typhimurium infections linked to pet hedgehogs that occurred during 2011–2013, 2018–2019 and July 2020 ((10,24–27)). These outbreaks were caused by a genetically similar strain of *S.* Typhimurium, as determined by whole genome sequencing (WGS), suggesting wide dissemination throughout the US pet hedgehog industry ((25–27)).

In October 2020, a Canadian investigation was initiated by the Public Health Agency of Canada (PHAC) and provincial public health officials when *S.* Typhimurium isolates from humans identified were genetically related by WGS to the US pet hedgehog outbreak ((26)). The objectives of the investigation are to identify the source of illness and risk factors for infection, determine if there is an epidemiologic link between the US and Canadian outbreaks, and implement public health interventions, including education and awareness activities.

## Method

### Overview

Following notification by the CDC on September 19, 2020, about an outbreak of *S*. Typhimurium infections linked to contact with pet hedgehogs ((25)), genetically related Canadian isolates were identified through PulseNet Canada (PNC) ((28)). The Canadian outbreak investigation began on October 28, 2020, with the objective of describing *S.* Typhimurium outbreak cases, and identifying and tracing the source of the outbreak.

### Outbreak detection and case identification

Since salmonellosis is a notifiable disease in Canada, clinical laboratories send *Salmonella* spp. isolates to provincial public health laboratories or to the National Microbiology Laboratory for WGS-based subtyping (implemented in 2017) ((29)). The PNC national database team at the National Microbiology Laboratory analyzes all Canadian WGS data in a centralized BioNumerics v7.6 (Applied Maths) database ((30)). Multi-jurisdictional clusters of *S.* Typhimurium were identified using a threshold of at least three *S.* Typhimurium isolates related within 0–10 whole genome multi-locus sequence typing (wgMLST) allele differences where two of three isolates are within five wgMLST alleles. All three isolates must have isolation dates within the last 60 days and at least one must be clinical. Allele ranges may expand during an investigation based on available laboratory, epidemiologic and other relevant evidence. Once a cluster is identified, PNC assigns a cluster code, and isolates subsequently identified as genetically related are added to the WGS cluster. Epidemiologists at CDC and PHAC regularly communicate regarding investigations of interest to both countries. As a result, representative isolates from the US investigation were used to search for matching Canadian isolates in the PNC database.

### Case definition

The case definition included Canadian residents or visitors to Canada with laboratory confirmation of *S.* Typhimurium matching the outbreak cluster by WGS with symptom onset, specimen collection, or isolation date on or after December 1, 2019. Cases were related within 0–46 wgMLST allele differences, which was supported by both epidemiologic and trace back data. As the investigation progressed, genetically related historical clinical isolates from cases with a symptom onset, specimen collection, or isolation date on or after June 1, 2017, were added to the investigation.

### Epidemiologic and trace back investigation

Cases with laboratory-confirmed *Salmonella* infections were routinely interviewed by local or regional public health authorities in most jurisdictions. The questionnaires captured exposure information for the seven days prior to symptom onset and generally cover clinical, travel, food and other risk factors including animal exposures. Consent for future follow-up was gathered at the time of interview.

Information was collected from initial interviews, and cases were re-interviewed by PHAC or individual provinces with a questionnaire focused on hedgehog exposures, which included the following queries:

- Where hedgehog exposure occurred (i.e. home, relative/friend residence, pet store)
- Where and when hedgehogs were purchased
- Type of contact with the hedgehog (i.e. direct contact such as holding, kissing and feeding the hedgehog, or indirect contact, such as being in a household where hedgehogs are kept, or contact with the hedgehog environment and/or enclosure)
- Type of food the hedgehog consumed
- If the hedgehog appeared sick
- Cleaning practices (i.e. bathing the hedgehog and cleaning supplies)
- Other animal husbandry practices implemented (i.e. disinfection, hand washing and isolation of sick or newly obtained hedgehogs)

Interviews with identified hedgehog suppliers (which included pet stores, wholesalers and breeders) collected details on facility husbandry practices, herd health history, *Salmonella* precaution protocols and client education practices. Data collection also allowed to determine if a common supplier was associated with outbreak cases.

### Epidemiologic and statistical analyses

The proportions of sick people who reported any animal contact and contact with hedgehogs specifically were compared with corresponding reference values from the Foodbook study, a population-based study of Canadians’ exposure to food, animals and water over a seven-day period ((31)). Exact probability testing was used to measure the statistical significance of the proportion of cases who reported animal contact compared to Foodbook reference values.

### Laboratory investigation

Environmental and hedgehog fecal samples were collected from cases’ homes and hedgehog suppliers’ premises. Samples were submitted to provincial public health laboratories for WGS, which was performed according to the current PNC protocol. Briefly, genomic DNA was extracted using the DNeasy blood and tissue kit (Qiagen) or Epicentre MasterPure Complete DNA and RNA Purification Kit (Mandel). Libraries were prepared using the Nextera XT library prep kit (Illumina) and sequenced using the Illumina MiSeq platform (Illumina), using either V2 or V3 chemistry to achieve an average genome coverage of greater than or equal to 40x. The analysis of WGS data was done using the *Salmonella* wgMLST schema within the BioNumerics v7.6 (BioMerieux) platform. A dendrogram was constructed with BioNumerics v7.6 using a categorical (values) similarity coefficient and an unweighted pair group method with arithmetic mean (UPGMA) clustering algorithm. The UPGMA is a hierarchical clustering method used to generate a dendrogram to visualize isolate relatedness; it allows for analyses to be rapidly updated as isolates are added during the course of an investigation.

## Results

### Epidemiological investigation

**Figure 1:**
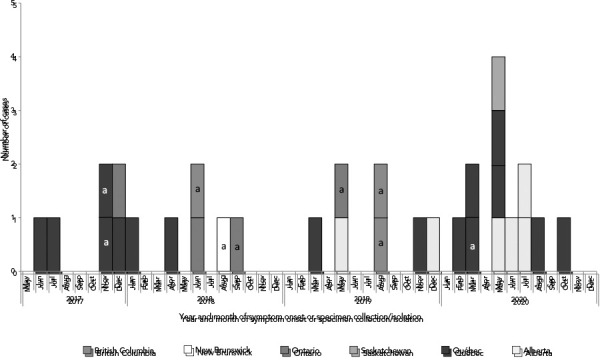
Number of cases with the outbreak strain of *Salmonella* Typhimurium by province and illness onset or specimen collection date (n=31) Note: The a indicates isolate for which only specimen collection or isolation date was available

A total of 31 cases were identified in six provinces (British Columbia [BC]=3, Alberta [AB]=6, Saskatchewan [SK]=1, Ontario [ON]=4, Québec [QC]=16 and New Brunswick [NB]=1). Symptom onset or specimen collection or isolation dates ranged from June 1, 2017, to October 15, 2020 (**Figure 1**).

Cases ranged in age from four months to 79 years with a median of 20 years. Thirty-two percent (n=10/31) were children aged 10 years of age or younger; of these, seven (70%) were two years of age or younger. Fifty-two percent of cases were female. Four of eight (50%) cases with available information were hospitalized and no deaths were reported (**Table 1**).

**Table 1:** Characteristics of persons infected with the outbreak strain of *Salmonella* Typhimurium (n=31)

Characteristics	Number of cases	Total cases	%
Age			
2 years of age or younger	7	31	23
3–10 years	3	31	10
11–20 years	6	31	19
21–50 years	9	31	29
Older than 50 years	6	31	19
Sex			
Female	16	31	52
Outcome			
Hospitalizations	4	8	50
Death	0	31	0

Animal exposure information was available for 26 of 31 (84%) cases. The proportion of cases who reported animal or pocket pet contact was significantly higher (*p*<0.001) than the general population when compared using the Foodbook study (**Table 2**). Nineteen cases reported exposure to pocket pets, all of which were hedgehogs. Fifteen reported direct contact with a hedgehog and three reported indirect contact (**Table 3**). Most cases reported bathing their hedgehog and cleaning their supplies in a sink or tub also used for other purposes, and three cases reported allowing their hedgehog to roam free in the home; all potential routes of indirect transmission. No commonalities were observed among hedgehog diets.

**Table 2:** Summary of animal contact and pocket pet exposures among persons infected with the outbreak strain of *Salmonella* Typhimurium (n=26), compared with population-based reference values^a^

Exposure	Number of cases	% of cases	Reference value (%) (Canada)	*p*-value
Animal contact	26/26	100	63.4	<0.001
Pocket pets^b^	19/26	73	3.4	<0.001

^a^ Murray R *et al.* Canadian consumer food safety practices and knowledge: Foodbook study. J Food Protect. 2017;80 (10):1711-8

^b^ Pocket pets include mice, rats, gerbils, hamsters, guinea pigs, ferrets and hedgehogs

**Table 3:** Description of hedgehog-related exposures and interactions among persons infected with the outbreak strain of *Salmonella* Typhimurium

Exposures or interactions	Number of cases n/N^a^	% of cases
Type of hedgehog exposure		
Direct contact	15/18	83
Touching and/or holding	10/15	67
Indirect contact	3/18	17
History of hedgehog illness		
Ill prior to case symptom onset	3/16	19
Length of hedgehog ownership prior to case illness		
One month or less	7/15	47
Two to three months	6/15	40
Approximately one year	2/15	13
Hedgehog hygiene practices		
Allowed to roam free around the house	3/16	19
Bathed and cleaned supplies in case’s home tub or sink in the kitchen, bathroom, or laundry	11/14	79
Bathed and cleaned supplies in case’s home in sink or bin designated for this purpose	3/14	21
Hedgehog diet^b^		
Kitten/cat kibble	14/19	74
Mealworms	11/19	58
Fruits/vegetables	1/19	5

^a^ Denominators differ as a result of missing data

^b^ Categories not mutually exclusive

### Traceback investigation

**Figure 2:**
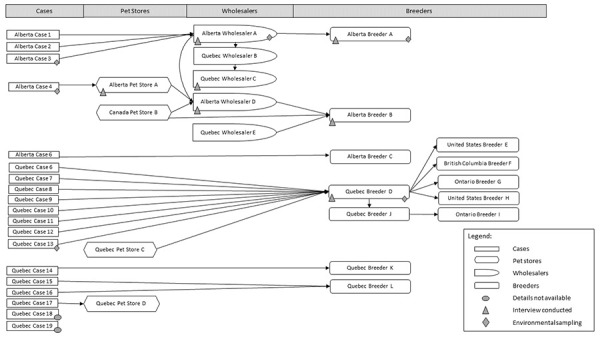
Traceback network diagram of hedgehogs associated with sick persons infected with the outbreak strain of *Salmonella* Typhimurium^a^ ^a^ All cases with hedgehog exposures are shown in rectangles. All pet stores (depicted by hexagons) and wholesalers (depicted by half circles) in this diagram were located in Alberta and Québec, Canada. The breeders (depicted by rounded rectangles) were located in Alberta, Québec, British Columbia and Ontario, Canada and in the United States. Filled circles depict cases where further hedgehog information was not available. The triangles depict the location of hedgehog suppliers that were interviewed, and the stars depict the locations where environmental sampling was performed. Arrows illustrate the reported links by cases or suppliers

Hedgehog suppliers were identified for 21/23 (91%) cases: 4 pet stores; 5 wholesalers; and 12 breeders (**Figure 2**). Although no single source was identified, there were common suppliers reported and one direct link identified between the Canadian and US outbreak investigations, as one breeder located in the US was reported in both investigations (Figure 2). Six suppliers were interviewed and all reported being aware that hedgehogs can carry *Salmonella* and take precautions to prevent zoonotic transmission.

### Laboratory investigation

Environmental samples from hedgehog habitats and fecal samples were collected from three cases’ homes, one wholesaler and two breeders. One hedgehog stool sample collected from a case’s home in QC tested positive and was genetically related to the outbreak strain based on WGS. All other samples were negative for *Salmonella*. An additional hedgehog stool isolate genetically related to the outbreak by WGS was identified from a sample collected in July 2020 during routine quarantine exams at a QC zoo; however, the supplier of this hedgehog was a breeder in QC with no identified connection to the hedgehog suppliers reported by cases (*personal communication Ministère des Forêts, de la Faune et des Parcs*).

**Figure 3:**
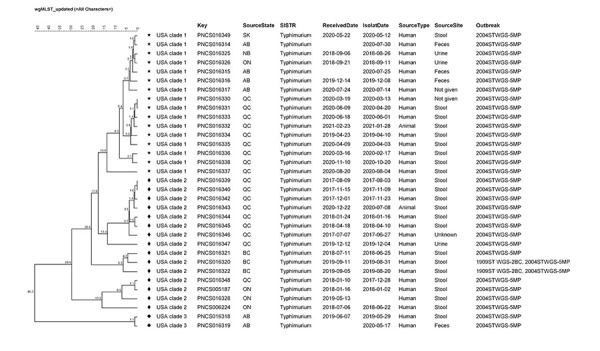
Relatedness of outbreak-associated isolates by whole genome sequencing multi-locus sequence typing^a^ ^a^ Unweighted pair group method with arithmetic mean (UPGMA) dendogram of whole genome multilocus sequence typing (wgMLST) results for human and animal isolates included in the investigation, generated using BioNumerics v7.6 (Applied Maths)

The 33 isolates grouped together with 0–46 wgMLST allele differences, and were genetically related to a concurrent US investigation associated with hedgehogs. In the US investigation, isolates were grouped into three clades based on their genetic profiles; Canadian isolates were genetically related to all three clades from the US (**Figure 3**) ((26,27)). Notably, nine isolates from QC (including one animal) grouped together in clade 1 and were linked to a specific breeder. A pairwise comparison between the isolate of QC case 13 and their hedgehog’s isolate showed they were within three wgMLST allele differences of each other. Four AB isolates were also in clade 1, and grouped more tightly with isolates from SK, NB and ON than the QC isolates. Nine isolates from QC (including one animal), along with isolates from ON and BC, were in clade 2, and two isolates from AB were in clade 3.

### Public health response and interventions

A Public Health Notice was issued by PHAC on November 6, 2020, to notify the public about the outbreak and share prevention tips on how to safely interact with pet hedgehogs ((7,24)). Teleconferences were held by PHAC and CDC with Canadian and US hedgehog industry members to notify them about the outbreak and provide key prevention principles to help reduce the risk of disease transmission from hedgehogs to humans ((13,14)).

## Discussion

This is the second *Salmonella* outbreak linked to pet hedgehogs in Canada, and the first caused by *S.* Typhimurium ((18)). The investigation identified 31 cases in six provinces, from June 2017 to October 2020. With 73% of cases reporting exposure to hedgehogs, the epidemiologic information provided strong evidence to the source of the outbreak, further strengthened by laboratory and trace back investigations. The investigation revealed a large, interconnected network of hedgehog suppliers, with some cases’ hedgehogs linked to common suppliers, but no single source of the infections. Results of this outbreak investigation emphasize the risk of *Salmonella* transmission from pet hedgehogs to humans, as previously described ((10,18)).

As was the case in this outbreak, children are often disproportionately affected in pet-related outbreaks ((26,32–37)). Young children have higher risk of developing more severe salmonellosis, are more likely to get tested, and often more likely to be exposed through both increased contact with pets and less vigilant hand washing ((5,34,35,38–41)). Although most cases reported direct contact, only indirect contact was reported by 17% of cases, including two one-year-old children. This speaks to the difficulty in preventing cross-contamination in homes. It is not recommended to keep hedgehogs in households with children younger than or five years old and strict hygiene practices should be adopted around these pets ((7)).

The WGS analysis, epidemiologic and trace back evidence helped inform the case definition and characterize distribution of the outbreak strain of *S.* Typhimurium. The search for highly related cases in previous years was limited because WGS analysis of *Salmonella* isolates began in 2017. Nonetheless, cases from 2017 to 2019 were identified, indicating the presence of this strain in Canada since at least 2017. This strain also caused reoccurring outbreaks of human infections linked to contact with pet hedgehogs in the US as far back as 2011–2013, suggesting its persistence in the hedgehog industry ((6,26,27)). One direct link to the concurrent US outbreak was identified during the trace back investigation. A hedgehog breeder in the US was connected to QC “Breeder D”, identified as a common source by eight cases, including QC case 13 whose hedgehog’s isolate was genetically related to the outbreak. This same US breeder was also linked to other US suppliers identified as sources of hedgehogs of cases in the US investigation ((26)).

The expansion of the case definition to include older samples from 2017 to 2019 helped to demonstrate the ongoing persistence of this strain in hedgehogs in Canada. The older samples may also reflect a baseline of sporadic infections for this *S.* Typhimurium strain, of 6–7 cases per year, with 0–2 cases per month and 0–5 months between cases. The original outbreak case definition, which includes cases from December 1, 2019, or after, would therefore be more accurate, since between then and October 2020 the number and frequency of cases exceeded the baseline incidence. Cases matching the outbreak strain then decreased to expected monthly baseline incidence, and the outbreak was declared over on December 18, 2020. Since this strain is an ongoing issue in hedgehogs in the US ((26)), and based on epidemiologic information gathered through this outbreak, it can be confirmed that sporadic cases occurred and might continue to occur in Canada with an occasional increase in incidence, potentially signalling an outbreak event. The use of WGS will be useful to distinguish between outbreak-associated and sporadic illnesses. In this outbreak, the US reporting on their outbreak and associated early signal of hedgehog contact also resulted in a strengthened rationale for additional epidemiological follow-up on genetically related Canadian cases and highlighted a potential source for the illnesses identified.

Isolates from cases whose hedgehogs were traced back to a common source were found to be closely related genetically. For example, isolates from the eight cases and one hedgehog associated with QC “Breeder D” differed by 16 wgMLST alleles or fewer, and the four isolates from cases associated with AB “Wholesaler A” were within four allele differences, compared with 46 alleles difference for all outbreak-associated isolates. Other outbreak-associated isolates were closely related genetically too but could not be traced to a common hedgehog source, with cases’ residences spread geographically across Canada and illness onset dates spanning a wide temporal range. The proportion of cases by province also varied over time: cases from QC (52% of all cases) were observed throughout 2017–2020 while cases from AB were observed in 2019–2020, suggesting a more recent introduction of the outbreak strain in AB. These findings might be explained by the interconnected and dynamic hedgehog distribution network, but would require further investigation to elucidate.

### Limitations

Limitations to the investigation include 1) the inability to re-interview all cases with the focused questionnaire as some were retrospectively linked through WGS and 2) the absence of hedgehog exposure reported by some cases. For the latter, it is possible these cases had unknown indirect exposure to hedgehogs. The inability to interview more hedgehog suppliers also limited full understanding of the interconnectedness in the supplier network which could have provided more details of potential transmission pathways.

## Conclusion

This investigation benefited from strong collaboration between Canadian partners in public and animal health at the provincial and federal level, the pet industry including Pet Industry Joint Advisory Council of Canada and the CDC. Communication between these groups and to the public aimed to increase awareness and provided education regarding the risk of *Salmonella* infection from hedgehogs and proper hygienic practices, with the goal of preventing further disease transmission.

Although the carriage rates and transmission dynamics in the pet hedgehog industry are not well characterized, extrapolation from rodent models indicates that *Salmonella* carriage may be persistent and heterogeneous, with the majority of transmission occurring through heavily infected super spreaders ((42)). During this investigation, members of the hedgehog industry expressed knowledge of *Salmonella* transmission prevention, yet one breeder reported treating all their hedgehogs with antibiotics upon hearing of the outbreak. Antibiotic-induced alterations in the intestinal microbiota are thought to increase the likelihood of colonization and shedding; antibiotic treatment is therefore contraindicated in non-clinical cases ((14,42,43)). Collaboration with the pet industry is needed to better understand transmission dynamics and target interventions to reduce levels of infection and transmission rates. The industry and its clients should be educated on the harms of indiscriminate antibiotic use, which potentially leads to more transmission, and selection for antibiotic resistant strains.

The high proportion of young children among cases in this outbreak emphasizes the importance of providing potential small pet owners the educational materials necessary to make informed decisions about pet choices and to implement safety precautions. Anecdotal reports suggest an increase in pet ownership during the coronavirus disease 2019 pandemic ((44,45)), which may include small pets like hedgehogs. While recognizing the benefits of having a pet, this outbreak of *S.* Typhimurium is a timely reminder of the importance of *Salmonella* awareness and education among suppliers and owners of small pets, to prevent disease transmission.
